# Enhanced multi-object detection in brachial plexus ultrasound images using transformer-based semantic and spatial feature fusion

**DOI:** 10.1186/s42492-026-00236-y

**Published:** 2026-11-13

**Authors:** Hua Wei, Xin Gao, Yuhao Zhong, Yi Ding, Ting Han

**Affiliations:** 1 School of Design, Shanghai Jiaotong University, Shanghai, 200240 China; 2 State Key Laboratory of Media Convergence Production Technology and Systems, Xinhua News Agency, Beijing, 100803 China; 3 Network and Data Security Key Laboratory of Sichuan Province, School of Information and Software Engineering, University of Electronic Science and Technology of China, Chengdu, 610054 Sichuan China; 4 YIBIN GREAT Technology Co., Ltd, Yibin, 644000 Sichuan China

**Keywords:** Brachial plexus ultrasound, Object detection, Transformer, Semantic and spatial feature fusion, Self-distillation

## Abstract

Advances in medical imaging have led to the widespread use of ultrasound for diagnosing and treating peripheral nerve blocks. However, challenges such as speckle noise and low spatial resolution hinder the accurate identification of neural structures. To address these issues, a model named transformer-based detection with semantic and spatial feature fusion (TDSS) was introduced, integrating spatial edge adaptive enhancement, multi-scale semantic fusion, and spatial distillation-assisted detection modules. These components improve edge perception, multi-scale semantic reasoning, and localization accuracy. Experiments on the Ultrasound Brachial Plexus Dataset demonstrated the superior performance of the model: TDSS achieved an AP of 0.407 for nerve detection and an overall mAP of 0.583, providing robust support for clinical medical image detection. The source code for the proposed TDSS framework is publicly available at https://doi.org/10.5281/zenodo.18824199.

## Introduction

Ultrasound imaging is increasingly used in medical diagnostics, particularly for performing peripheral nerve blocks (PNBs). PNBs involve injecting a local anesthetic around specific nerves to numb the surgical or treatment area. This anesthetic method significantly reduces postoperative pain, shortens recovery time, and enhances patient comfort. Moreover, using this technique, anesthesiologists, under ultrasound guidance, can identify and locate target neuroanatomical structures such as nerves, arteries, veins, muscles, and surrounding tissues, while also monitoring the needle tip and drug diffusion. Currently, positioning of the brachial plexus and its surrounding tissues largely relies on manual efforts by physicians, a process that is time-consuming and requires extensive experience and anatomical knowledge. Leveraging deep learning for multiobject detection, including veins, arteries, nerves, and muscle tissue, can significantly aid the precise detection of nerve structures. This approach can substantially reduce the workload of physicians, enhance the accuracy of anatomical positioning, and improve surgical efficiency. Multi-object detection is the cornerstone of computer vision. In natural images, traditional convolution-based algorithms such as Mask R-CNN and Swin transformer have shown significant advances, and transformer-based end-to-end object detection frameworks have recently made substantial progress. For instance, DINO (DETR with Improved deNoising anchor boxes) surpasses traditional convolution-based object detection frameworks in multiple detection tasks. Xu et al. [1] developed an enhanced Faster R-CNN model for detecting tuberculosis in CT scans, and Ding et al. [2] introduced a cross-modal transfer learning cascading framework (CM-TranCaF) for segmenting cardiac MRI images. In contrast to natural images, ultrasound images feature prominent speckle noise and low spatial resolution, making it particularly challenging to precisely delineate the subtle structures of the brachial plexus [3, 4]. Inherent visual ambiguity arises because the acoustic impedance differences between the target neural tissues and background structures are minimal, which obscures the semantic boundaries. This further complicates the annotation of extensive medical image datasets, thereby hampering the acquisition of large-scale accurately annotated datasets. Moreover, pretraining techniques commonly used in natural image object detection often fail to deliver optimal results in medical imaging tasks because of the significant domain gap between natural textures and ultrasonic speckle patterns.

**Fig. 1:**
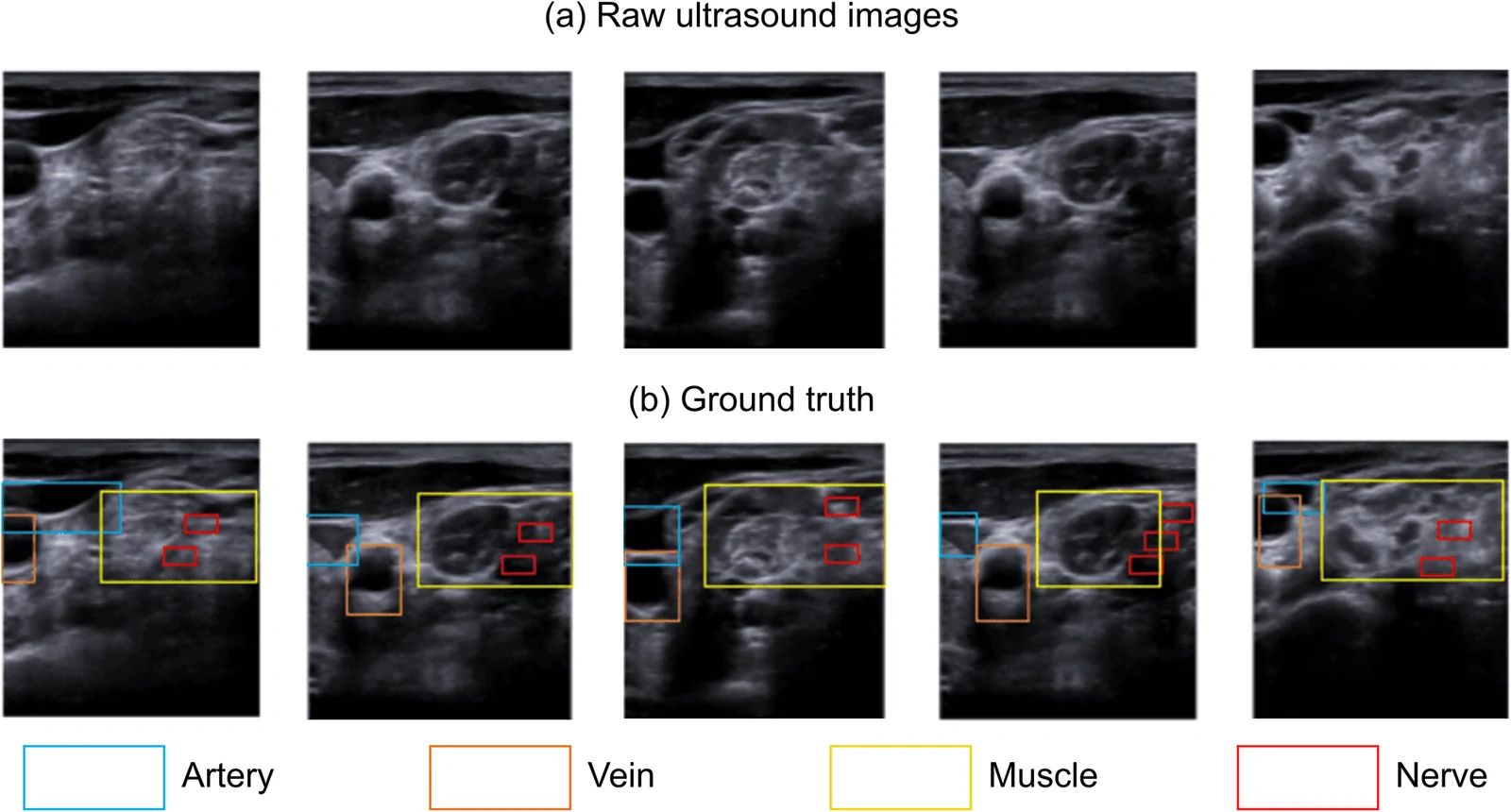
Original images and ground truth values of multiple brachial plexus ultrasound images

Representative brachial plexus ultrasound images and their ground-truth annotations are shown in Fig. 1.

To address these challenges, transformer-based detection with semantic and spatial feature fusion (TDSS), a framework composed of three plug-and-play modules, each of which can be independently integrated into existing detection pipelines without extensive modifications, was proposed: The spatial edge adaptive enhancement (SEAE) module enhances the model’s ability to recognize target structures by leveraging edge-detection algorithms inspired by biological vision [5], which are highly sensitive to edge features and less sensitive to nontarget background features. This module not only focuses on local edge details but also captures global contextual information by analyzing imaging differences across various biological structures. The multi-scale semantic fusion (MSF) module addresses the complexity and diversity of the ultrasound images by integrating features across multiple scales. It constructs a pyramid of feature maps at different resolutions, enabling the model to simultaneously process both the detailed and global features of the images. The MSF module, by employing both channel and spatial cross-attention mechanisms, extracts and synthesizes information across these scales, thereby significantly enhancing the model’s semantic interpretation capabilities. Finally, the spatial distillation-assisted detection (SDAD) module uses the classification outcomes to refine the localization of the target structures. It employs self-distillation techniques to optimize the bounding box positions even under noisy imaging conditions, thereby improving the precision of the detection tasks. Through a combination of spatial positional self-distillation and multi-instance learning, the SDAD module ensures the selection of the most accurate proposals for object detection, thereby effectively enhancing the overall detection performance. The primary contributions of this study are as follows:

1. An integrated framework specifically designed for multi-object detection in brachial plexus ultrasound images was proposed to effectively mitigate issues related to anatomical variability and inherent noise.
2. Three plug-and-play modules were introduced: the SEAE module for edge delineation in the presence of noise, the MSF module for multi-scale semantic integration, and the SDAD module for refining localization through self-distillation.
3. Through extensive experiments on the Ultrasound Brachial Plexus Dataset (UBPD), the proposed framework demonstrated superior performance compared with state-of-the-art methods, particularly in detecting complex neural structures.

### Object detection applied in medical images

Traditional object detection models are typically categorized into two-stage and single-stage paradigms. Two-stage models, such as Mask R-CNN [6] and Cascade R-CNN [7], utilize a region proposal network to generate object candidates that are subsequently refined. While accurate, their serialized nature often limits the inference speed. Conversely, single-stage models such as the YOLO series prioritize real-time efficiency [8].

The introduction of transformer architecture, notably DETR [9], marked a paradigm shift toward end-to-end detection by eliminating manual components such as non-maximum suppression. Ongoing research has significantly enhanced these methods [10], paving the way for a competitive advantage in medical imaging [11]. Specialized architectures, such as the dual encoding-decoding X-Net [12], have demonstrated the effectiveness of integrating global contextual features with localized representations. Recent advancements continue to expand these capabilities; for instance, Zhang and Tian [13] proposed a mutual-attention-based transformer for image-anomaly detection, while Dai and Yao [14] introduced defocus-tolerant view synthesis using Gaussian splatting to enhance visual consistency. Furthermore, Cai and Zhou [15] leveraged panoramic models for real-time camera calibration, highlighting the importance of a robust feature representation in evolving environments.

In ultrasound analysis, a survey by Marques et al. [16] underscored the value of edge-aware strategies to navigate heavy speckle noise. This aligns with recent large-scale clinical studies, such as the deep learning system developed by Li et al. [17] for detecting systemic conditions from retinal scans and AI-driven diagnostic strategies for myopic maculopathy reported by Qian et al. [18], both of which emphasize the necessity of high-precision feature extraction in noise-prone medical data.

### Attention mechanism

Attention mechanisms emulate biological visual perception by dynamically adjusting weights based on input features [19]. In deep networks, channel attention models such as SENet [20], ECA-Net [21], and FcaNet [22] recalibrate the importance of different channels to enrich information.

Spatial attention highlights specific regions, and GENet [23] and non-local networks [24] address long-distance dependencies. A vision transformer (ViT) [25] further enables models to concentrate on pertinent areas while discarding background noise [26]. Crucially, for low-quality medical scans, structure-aware transformer-based attention fusion networks, such as Sat-net proposed by Wen et al. [27], have proven to be essential for enhancing feature clarity and detail. In brachial plexus ultrasound imaging, these mechanisms allow the model to allocate resources to subtle or indistinct neural structures by mimicking the human focus.

### Self-distillation

Self-distillation derives guidance knowledge internally within a network [28] to boost accuracy without the complex design of external teacher architectures [29, 30]. Initially introduced in LGD [29], this method integrates label features with the model’s feature map to capture complex inter-object relationships. In the context of brachial plexus ultrasound images, employing self-distillation facilitates a more efficient feature extraction strategy, aiding in the identification of anatomical structures through richer guidance and mitigating the impact of inconsistent boundaries in noisy datasets.

## Methods

### Overview

**Fig. 2:**
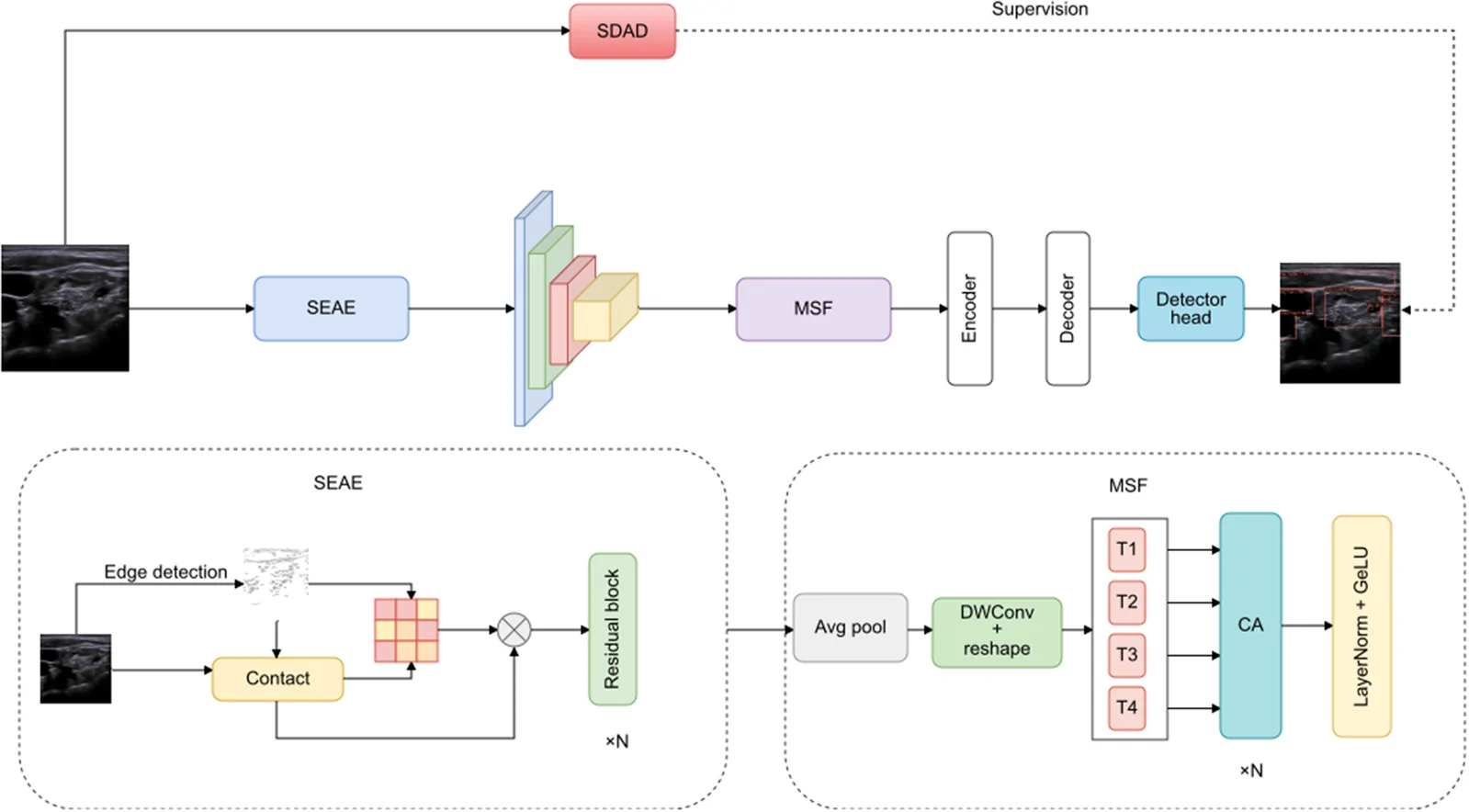
Overall architecture of the transformer-based detection with semantic and spatial feature fusion. It contains SEAE, MSF, SDAD, and the detector head. The TDSS network leverages edge-sensitive feature extraction, multi-scale semantic fusion, a transformer based encoder-decoder structure, and spatial distillation-assisted detection to enhance the accuracy of object detection in ultrasound images. TDSS: Transformer-based detection with semantic and spatial feature fusion; SEAE: Spatial edge adaptive enhancement; MSF: Multi-scale semantic fusion; SDAD: Spatial distillation-assisted detection

The TDSS method, an advanced object detection framework designed specifically to enhance the detection of multiple objects within brachial plexus ultrasound imagery, was introduced. This method leverages a comprehensive end-to-end detection approach, facilitated by three intricately designed modules illustrated in Fig. 2.

Initially, the network processes an ultrasound image with dimensions H *×* W *×* C. The SEAE module leverages the sensitivity of biological vision to edge features and extracts these features from a feature map using advanced edge-detection techniques. This process generates a high-quality attention distribution map denoted by *A*, which, in turn, produces an effective mask *M*. This mask significantly improves the quality of the feature map *F*, thereby enhancing the network’s capability to accurately distinguish anatomical targets from background speckle noise.

After backbone feature extraction, the MSF constructs a multi-scale pyramid structure, integrates feature maps $E_1,E_2,\ldots,E_n$ of different resolutions, and uses channel cross-attention and spatial cross-attention to extract global channel and spatial features to enhance the model’s ability to understand and express the dependence between features at different scales. The network follows the encoder-decoder structure of the transformer-based object detection framework. In the transformer encoder, global features are further learned through self-attention, and the enhanced feature map is converted into the final detection result, including the position and category of the bounding box, through the transformer decoder and detection head.

At the same time, the SDAD module uses the classification results as a guidance signal for positioning, generating high-quality proposal packages through self-distillation and neighborhood sampling; it then performs fine-grained corrections through the regressor, significantly improving proposal quality. The SDAD module further combines category and spatial information to evaluate the spatial relationship between each proposal and target more accurately, completing the entire process from the input images to the output detection results.

Through this comprehensive method, TDSS can effectively deal with noise interference in ultrasound images and improve the accuracy and robustness of object detection.

### SEAE

For each input image, the Canny operator was applied to calculate the gradients in both horizontal and vertical directions, thereby deriving an edge map $X_e$ following Canny extraction. Subsequently, $X_e$ was combined with the original image *X* to form a composite input, denoted as $X_c$, which integrates the original and the edge features.

The Canny operator uses lower and upper thresholds of 40 and 120, respectively. The resulting edge map is not used directly as the final anatomical boundary or detection result. Instead, it serves as a low-level structural prior inside SEAE. After edge extraction, the edge map is further processed by convolution, batch normalization, ReLU activation, feature fusion, and sigmoid attention gating before being integrated into the deep detection features. Therefore, the false edge responses caused by speckle noise are not directly converted into object predictions. During end-to-end training, edge responses that are inconsistent with annotated anatomical structures are suppressed by detection loss. In addition, Gaussian noise augmentation is adopted during training to improve robustness to ultrasound noise.

**Fig. 3:**
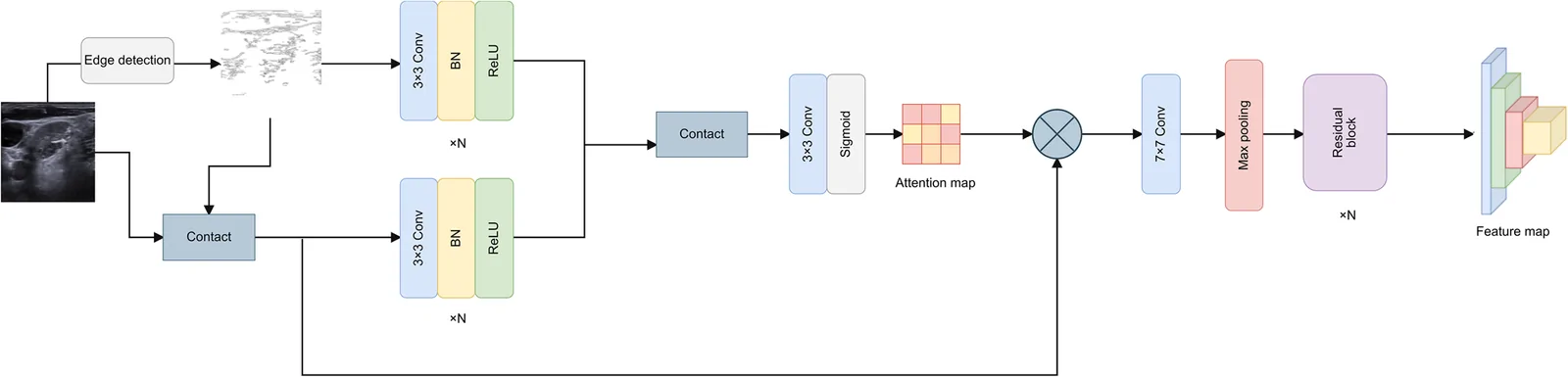
Structure of the spatial edge adaptive enhancement module. Spatial edge adaptive enhancement enhances edge feature extraction in brachial plexusultrasound images by combining Canny edge detection with convolutional processing, producing anattention map that highlights tissue edges, and utilizing ResNet-50 for multi-scale feature fusion toimprove model performance

The architecture of the SEAE module is shown in Fig. 3.

1 $$ \begin{aligned} X_e &= \mathrm{EdgeDetection}(X) \\ X_c &= \mathrm{concat}(X, X_e) \end{aligned}$$

To enhance the extraction of edge features from the target tissue in brachial plexus ultrasound images, a series of N-network blocks was employed. Each block comprises a convolutional layer with a 3 *×* 3 kernel size, followed by a batch normalization layer and a ReLU activation function, which are collectively utilized for the extraction of the feature map $F_e$.

2 $$ F_e = \mathrm{ReLU}(\mathrm{BN}(\mathrm{Conv}(X_e)))$$

During ultrasound imaging, the intensity of the reflected signals varies significantly among different tissues. To emphasize these variations and capture the global channel dependencies in the ultrasound image, the edge image $F_e$ was combined with the original image *X* to create $F_p$. This combined image is then processed through a network block consisting of a convolutional layer with a 3 *×* 3 kernel size, batch normalization layer, and ReLU layer. This processing step further maps the target features, resulting in hybrid feature map $F_c$.

3 $$ F_c = \mathrm{ReLU}(\mathrm{BN}(\mathrm{Conv}(X_c)))$$

The feature maps $F_e$ and $F_c$ were concatenated to facilitate information fusion, and the combined features were then passed through a convolutional layer. By merging the edge features $F_e$ and hybrid features $F_c$, the resulting feature $F_m$ was obtained. These features were processed using a sigmoid function to produce an attention map *A,* which effectively delineated the boundaries of the brachial plexus. The inner product of the attention map *A* and feature $F_p$ was then computed to generate the spatial edge feature map $F_b$, representing the processed original input image.

4 $$ \begin{aligned} \mathrm{A} &= \frac{1}{1 + e^{-F_m}} \\[2ex] F_b &= \sum_{i=1}^{H \times W} \mathrm{A}_{ji} F_p \end{aligned}$$

where *j* denotes the spatial position index in the attention map, and i indexes the spatial locations of the feature map.

In addition, a classical feature-extraction approach using the ResNet-50 architecture was employed. By stacking multiple residual blocks, multi-scale features *F* were generated and subsequently subjected to multi-scale feature fusion to enhance the model’s capability.

### MSF

**Fig. 4:**
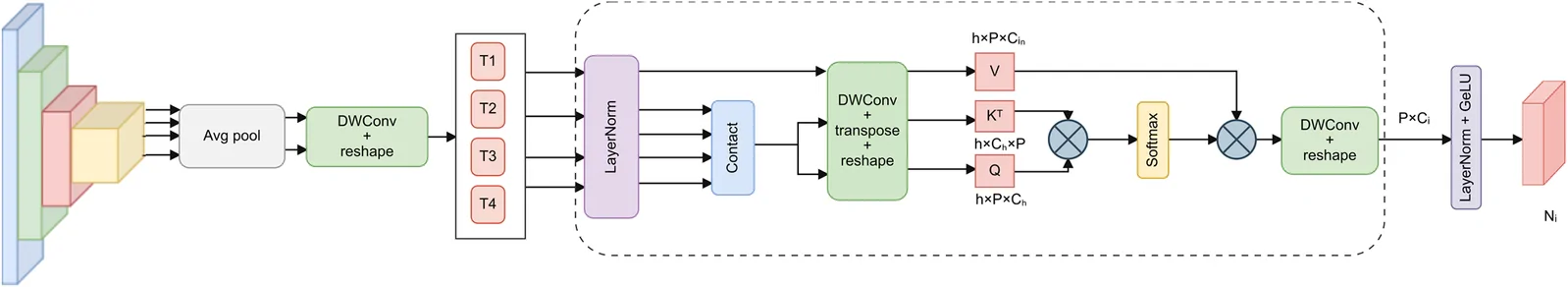
Structure of the multi-scale semantic fusion module. Multi-scale semantic fusion utilizes an advanced cross-attention mechanism andmulti-scale patch embedding to integrate and enhance spatial features across various scales, improvingmodel performance

In traditional multi-scale pyramid structures, cross-scale spatial features are typically extracted through simple interactions between feature maps at different scales. Inspired by this methodology, a multi-scale cross-attention module was introduced that leverages an advanced cross-attention mechanism to effectively capture spatial dependencies across multi-scale feature maps, facilitating the extraction of both global and local spatial information. The architecture of the MSF module is shown in Fig. 4.

The Swin transformer introduces padding, which enhances the efficiency of the transformer across various visual tasks. Utilizing a multi-scale patch-embedding method allows tokens to be generated at different scales. For a feature map $F_i$ with a scale $H_i\times W_i\times C_i$, the patch size $P_i$ is defined as $\frac{P}{2^{i-1}}$, where *i* ranges from 1 to *n*. Initially, average pooling was employed to extract the tokens, after which the linear projection was replaced with a 1 *×* 1 depthwise separable convolution (DWConv). This approach uniformly maps the feature map of each scale to an equivalent number of patches.

5 $$ T_i = \mathrm{DWConv}(\mathrm{Reshape}(\mathrm{AvgPool}(F_i)))$$

For the reshaped $T_i$, spatial features are extracted through layer normalization and cascading operations along the channel dimension. Specifically, *i* tokens undergo layer normalization and are cascaded along the channel dimension to form a linked token $T_c^{\prime}$ with dimensions *P× C* (where *C* denotes the total number of channels after concatenation). These tokens are then reshaped to serve as projected queries and keys, and each $T_i^\prime$ is used as a value.

6 $$ \begin{aligned} Q &= \mathrm{Reshape}(\mathrm{Transpose}(\mathrm{DWConv}(T_c^\prime))) \\ K^T &= \mathrm{Reshape}(\mathrm{Transpose}(\mathrm{DWConv}(T_c^\prime))) \\ V &= \mathrm{Reshape}(\mathrm{Transpose}(\mathrm{DWConv}(T_i^\prime))) \end{aligned}$$

$\mathbf{Q}\in\mathbb{R}^{P\times C}$,$\mathbf{K}\in\mathbb{R}^{P\times C}$,$\mathbf{V}_i\in\mathbb {R}^{P\times C_i}$ denote the projected query, key, and value, respectively. Using the softmax function and the dot product operation, the following multi-scale spatial attention was obtained:

7 $$ A_i = \mathrm{Softmax} \left( \frac{\mathbf{Q} \mathbf{K}^T}{\sqrt{d_k}} \right) \mathbf{V}_i$$

where $d_k$ denotes the channel dimension of the query/key vectors and is used as the scaling factor in scaled dot-product attention.

The output of the MSF module was projected using depthwise convolution, followed by the application of layer normalization and the Gaussian error linear unit activation function. This process yielded the final layer feature $N_i$, which served as the input for the subsequent encoder.

8 $$ N_i = \mathrm{GeLU}(\mathrm{LayerNorm}(\mathrm{Reshape}(\mathrm{Transpose}(\mathrm{DWConv}(A_i)))))$$

Similar evidence was reported by MSFSegNet [31] for liver ultrasound instance segmentation, which showed that fusing complementary features across multiple receptive fields markedly improved boundary delineation, a finding that supports the design philosophy of the proposed MSF block.

This spatial cross-attention mechanism not only integrates the spatial features of multi-scale feature maps, but also generates attention maps that capture the long-range dependencies between feature maps at various scales. Consequently, this approach significantly surpasses the performance of traditional multi-scale pyramid structures.

### SDAD

**Fig. 5:**
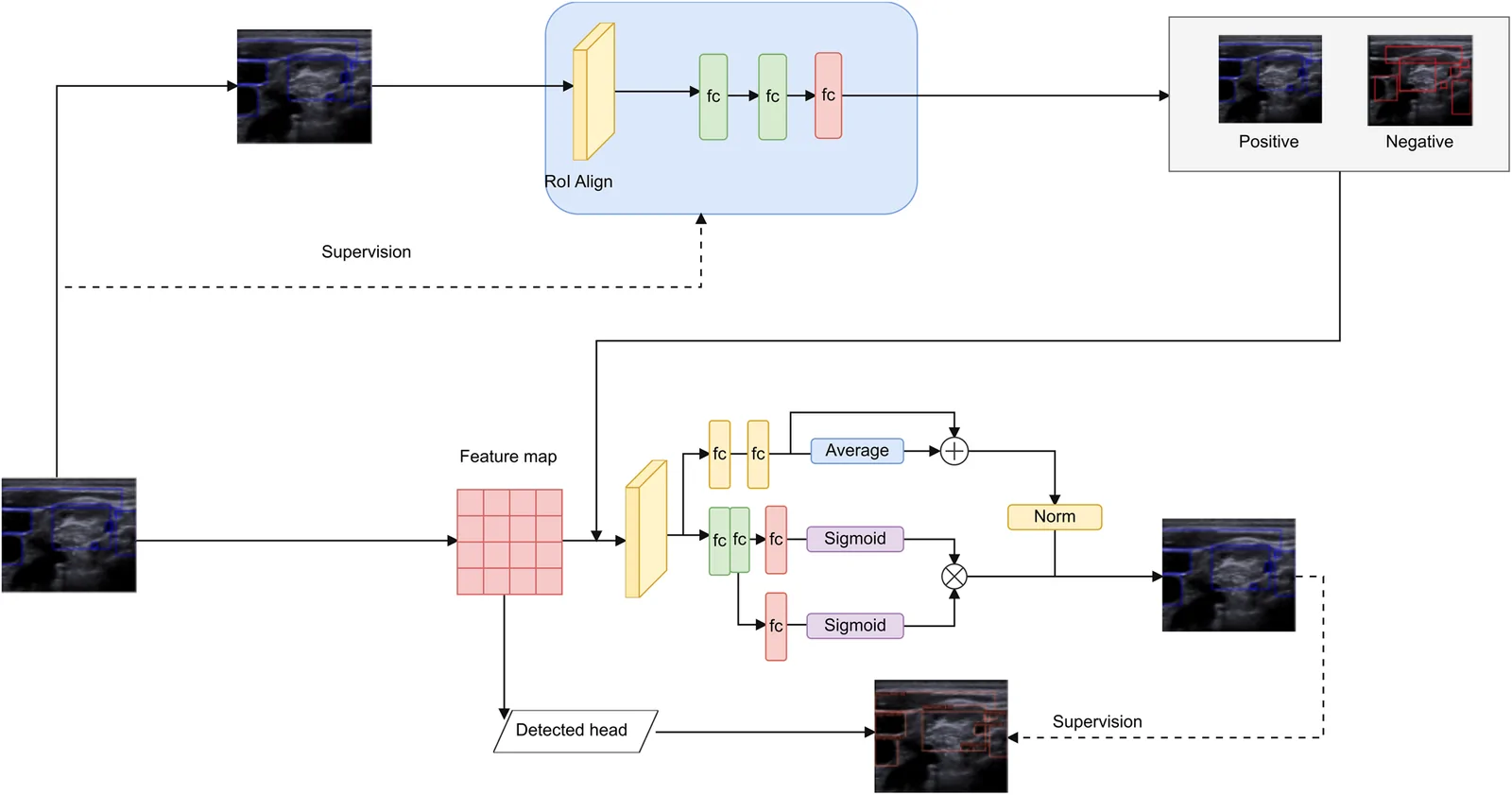
Structure of the spatial distillation-assisted detection module. Spatial distillation-assisted detection employs a self-distillation strategy utilizing multiple instance learning to construct object packages for proposal selection, followed by featureextraction and regression through RoI Align and fc layers

In ultrasound-image detection, accurate target localization is considerably more challenging than instance classification. To address this, a self-distillation strategy was implemented to refine the original bounding boxes of ultrasound images. Using multiple instance learning (MIL) [32], an object package was constructed for proposal selection within the detection box. The architecture of the SDAD module is shown in Fig. 5. The quality of these proposals critically influences the upper detection performance limit. For each bounding box *b*, its scale and aspect ratio, *s* and *v*, and its position, $$ o_x$$, $$o_y$$, are adjusted to obtain new proposals *b* = ($$b_x$$, $$b_y$$,$$ b_w$$,$$ b_h$$); Perturbations are applied as follows:

9 $$ \begin{aligned} b_w &= v \cdot s \cdot b_w^\ast \\ b_h &= (1/v) \cdot s \cdot b_h^\ast \\ b_x &= b_x^\ast + b_w \cdot o_x \\ b_y &= b_y^\ast + b_h \cdot o_y \end{aligned}$$

Taking the manually perturbed proposal package as the input, feature F is extracted through RoI Align and two fully shared (fc) layers. Subsequently, an additional fc layer $\widehat{f_c}$ is used for further regression.

Specifically, each proposal bag contains *P=8* proposals constructed by perturbing the scale, aspect ratio, and center offset of the original bounding box, thereby simulating possible localization variations around the anatomical target. The scale-jitter range is set to 0.20 and the center-jitter range is set to 0.10. The generated proposals are then processed through RoI Align and shared fc layers. Classification confidence and spatial consistency, including the predicted intersection over union (IoU) and center-distance constraints, are jointly used to select high-quality proposals for localization refinement; the top *K=3* proposals are retained for localization refinement. This design enables SDAD to reduce the bounding box drift under noisy and ambiguous ultrasound boundaries.

Under the supervision of the annotated boxes, the network acquires semantic and spatial knowledge from the dataset. To mitigate the impact of background noise, proposals with an IoU with the corresponding annotated box below a predefined threshold were selected as negative samples to assemble a negative sample package.

Subsequently, with the compiled positive and negative sample packages as inputs, the classification branch $f_{cls}$ is applied to the *F*, and the classification score $S^{c\ l\ s}$ is derived by applying a sigmoid function along the classification dimension *K*. Similarly, the instance-selection branch $f_{ins}$ is applied to *F*, and using the sigmoid function on *P* proposals, the instance score $S^{ins}$ is computed. The final proposal score, $\hat{S}$, is calculated by taking the Hadamard product of the classification and instance scores. Overall package score *S* is computed by summing the proposal scores across *P* proposal boxes.

10 $$ S = S^{cls} \odot S^{ins}, \quad \hat{S} = \sum_{p=1}^{P} [S]_p \in \mathbb{R}^K$$

To select proposals from the pool with high classification confidence and spatial proximity to the target, a spatial IoU and spatial distance (SISD) module was introduced to predict the IoU between proposals and their counterparts. Subsequently, by combining IoU and classification confidence, the top K proposals were selected. For proposed package B, features F are acquired through RoI Align and two fc layers. To encapsulate the characteristics of P proposals within proposal bag *b*, $F^+$ is computed by averaging the features of P proposals within proposal bag b, which is then added to the proposal features to derive the target-related features $F^\ast=F+F^+$. By utilizing a fully connected layer, the spatial confidence *C* is obtained, thereby facilitating filtering of the proposals.

### Loss function

In the TDSS framework, the overall loss function is defined as

11 $$ \mathcal{L} = \mathcal{L}_{\mathrm{Det}} + \alpha \mathcal{L}_{\mathrm{SDAD}}$$

where $\mathcal{L}_{\mathrm{Det}}$ represents the loss from the object-detection module and $\mathcal{L}_{\mathrm{SDAD}}$ denotes the loss from the SDAD module and *α* denotes a hyperparameter.

For the detector, L1 loss and generalized intersection over union loss were utilized for bounding box regression, while focal loss was utilized for classification. In the SDAD module, the loss comprises the loss from generating sample packages ($\mathcal{L}_{box}$) and instance selection loss ($\mathcal{L}_{select}$), defined as

12 $$ \begin{aligned} \mathcal{L}_{box} &= \frac{1}{P} \sum_{p=1}^{P} L_1([\mathcal{B}]_p, \hat{b}) \\ \mathcal{L}_{select} &= \mathrm{SmoothL}_1(U, T) \end{aligned}$$

$\left[\mathcal{B}\right]_p$ represents the generated instance package and $\hat{b}$ refers to the instance in the original input image. *U* is the predicted IoU and *T* is the IoU between the generated pseudo-label and $\hat{b}$. The SDAD loss $L_{SDAD}$ was then calculated as follows:

13 $$ \mathcal{L}_{\mathrm{SDAD}} = \mathcal{L}_{box} + \beta \mathcal{L}_{select}$$

where *β* denotes another hyperparameter.

In the default implementation, *α* is set to 0.35 to weight the overall SDAD loss, and *β* is set to 1.0 so that the box-generation and instance-selection terms have equal weights.

### Dataset details

In this study, the UBPD [33] was used as the primary benchmark. The UBPD dataset was specifically designed to detect the brachial plexus and surrounding anatomical structures to assist doctors in brachial plexus block procedures. The dataset, comprising data on 200 patients, was collected and annotated by experienced anesthesiologists. The ultrasound videos were acquired using two ultrasound systems, Siemens ACUSON NX3 Elite and Philips EPIQ5, which introduce device-level variation into the dataset. From each ultrasound video, 10–15 frames were randomly selected to ensure that each frame contained at least one region of interest. In total, the dataset comprised 2035 annotated images, each containing labels for key anatomical features such as arteries, veins, nerves, and muscle tissue.

For the experiments, the UBPD dataset was divided into training and testing sets based on patients to ensure no data leakage. The training set included 1835 images from 180 patients, whereas the test set comprised 200 images from 20 patients. Importantly, all patient data in the dataset were anonymized to protect patient privacy.

As shown in Table 1, the object-size distribution is highly imbalanced across anatomical categories. Nerve instances are dominated by small and medium objects, whereas muscle tissues mainly consist of large instances. This scale diversity motivated the design of the MSF module, which integrates multi-scale semantic features to improve the detection of anatomical structures of different sizes. This also indicates that improvement of the nerve AP is particularly important for ultrasound-guided brachial plexus localization.

**Table 1:** Object size distribution of anatomical categories in the UBPD dataset

Category	Total	Small	Ratio	Medium	Ratio	Large	Ratio
Artery	1381	1	0.07%	740	53.58%	640	46.34%
Vein	1276	18	1.41%	601	47.10%	657	51.49%
Nerve	4479	2006	44.79%	2471	55.17%	2	0.04%
Muscle tissue	3919	0	0.00%	130	3.32%	3789	96.68%

### Implementation details

The proposed TDSS model was trained using an end-to-end approach. Given that the ultrasound images in the dataset originated from different machines and varied in size, each image was resized to 640*×*640 pixels using bilinear interpolation. To improve model generalization, data augmentation techniques such as random flipping, scaling, and cropping were applied.

The detector follows the TDSS framework and was optimized using AdamW with a weight decay of $1\times 10^{-4}.$ Random horizontal flipping was applied with a probability of 0.5, random scaling was performed within [0.8, 1.2], random cropping was used to remove unnecessary image-boundary regions, and Gaussian noise augmentation was included to improve the robustness to ultrasound noise. All experiments were trained for 24 epochs using an initial learning rate of 0.01 and a batch size of 4. The learning rate was reduced to one-tenth of its initial value at the 16th epoch and to one-hundredth of its initial value at the 22nd epoch. The experiments were implemented using PyTorch and conducted on an NVIDIA A100 GPU using mixed-precision training. For the controlled comparisons shown in Tables 4, 5 and 6, the models were trained and evaluated on the same patient-level split of UBPD using the same input resolution and evaluation metrics. The feature-extraction and feature-fusion comparisons used Mask R-CNN with ResNet-50 and FPN as the common baseline, and the state-of-the-art methods in Table​ 2 followed their respective standard architectures.

**Table 2:** Performance comparison to different state-of-the-art methods on the test set of UBPD

Condition	Vein	Artery	Nerve	Muscle	mAP	AP50	AP75
Mask R-CNN (ICCV 2017)[6]	0.539	0.498	0.298	0.415	0.395	0.543	0.410
Cascade R-CNN (CVPR 2018)[7]	0.587	0.512	0.319	0.467	0.449	0.629	0.467
Dynamic R-CNN (ECCV 2020)[34]	0.611	0.565	0.329	0.459	0.454	0.636	0.466
Sparse R-CNN (CVPR 2021)[35]	0.586	0.502	0.216	0.427	0.418	0.585	0.399
DETR (ECCV 2020)[9]	0.375	0.287	0.092	0.285	0.298	0.418	0.283
ViTDet (ECCV 2022)[36]	0.652	0.637	0.344	0.472	0.529	0.741	0.555
DINO (ICLR 2023)[10]	0.704	0.682	0.392	0.515	0.569	0.797	0.583
YOLOv9 (CVPR 2024)[37]	0.695	0.670	0.380	0.510	0.564	0.790	0.575
Grounding DINO (ICLR 2024)[38]	0.700	0.675	0.385	0.520	0.570	0.795	0.580
RTMDet (CVPR 2023)[39]	0.705	0.680	0.390	0.515	0.573	0.800	0.585
MedSAM (Nature Comm. 2024)[40]	0.557	0.549	0.315	0.427	0.427	0.649	0.466
TDSS$^{\ast 1}$	0.711	**0.691**	0.399	0.527	0.578	0.799	0.590
TDSS	**0.712**	**0.691**	**0.407**	**0.531**	**0.583**	**0.804**	**0.593**

The best results are highlighted in bold. AP: Average precision; mAP: Mean average precision

**Table 3:** Performance of different feature extraction networks applied to the UBPD dataset

Condition	Vein	Artery	Nerve	Muscle	mAP
ResNet-50 [41]	0.539	0.498	0.298	0.415	0.395
VGG-19 [42]	0.541	0.508	0.307	0.426	0.403
VIT [25]	0.540	0.502	0.301	0.439	0.409
Swin [43]	0.561	**0.549**	0.312	0.469	0.426
Biformer [44]	**0.562**	**0.549**	**0.315**	**0.470**	**0.427**
SEAE	0.553	0.527	0.313	0.438	0.414

The best results are highlighted in bold. mAP: Mean average precision

**Table 4:** Performance of different multi-scale feature fusion networks applied to the UBPD dataset

Condition	Vein	Artery	Nerve	Muscle	mAP
FPN [45]	0.539	0.498	0.298	0.415	0.395
PANet [46]	0.540	0.515	0.305	0.433	0.403
FPT [47]	0.544	**0.523**	0.313	0.436	0.409
MSF	**0.545**	**0.523**	**0.317**	**0.439**	**0.416**

The best results are highlighted in bold. mAP: Mean average precision

**Table 5:** Performance evaluation of plug-and-play modules on the UBPD dataset

Condition	Vein	Artery	Nerve	Muscle	mAP
Mask R-CNN + ResNet-50 + FPN	0.539	0.498	0.298	0.415	0.395
Mask R-CNN + SEAE + FPN	0.553	0.527	0.313	0.438	0.414
Mask R-CNN + SEAE + MSF	0.545	0.523	0.317	0.439	0.416
Mask R-CNN + ResNet-50 + FPN + SDAD	0.541	0.525	0.303	0.424	0.402

mAP: Mean average precision

**Table 6:** Contribution of individual components to transformer-based detection with semantic and spatial feature fusion performance on UBPD dataset

Baseline	SEAE	MSF	SDAD	Vein	Artery	Nerve	Muscle	mAP	AP50	AP75
*\checkmark*				0.704	0.682	0.392	0.515	0.569	0.797	0.583
*\checkmark*	*\checkmark*			0.706	0.682	0.399	0.517	0.571	0.799	0.586
*\checkmark*	*\checkmark*	*\checkmark*		0.709	0.688	0.403	0.523	0.575	0.802	0.589
*\checkmark*	*\checkmark*	*\checkmark*	*\checkmark*	**0.712**	**0.691**	**0.407**	**0.531**	**0.583**	**0.804**	**0.593**

Note: A checkmark indicates that the corresponding baseline or proposed module is included in the modelconfiguration. The best results are highlighted in bold. SEAE: Spatial edge adaptive enhancement; MSF: Multi-scale semantic fusion; SDAD: Spatial distillation-assisted detection; AP: Average precision; mAP: Mean average precision

### Evaluation metric

The performance of the proposed model was evaluated using standard average precision (AP) scores at various IoU thresholds. AP scores measure the detection accuracy across four categories: arteries, veins, nerves, and muscle tissues. The mean average precision (mAP) metric aggregates AP across all categories, providing a single performance measure. IoU thresholds determine whether the predicted bounding boxes are classified as true or false positives. Specifically, AP50 refers to the AP at an IoU threshold of 0.5, and AP75 measures the precision at a higher threshold of 0.75. Additionally, the mAP score is computed over an IoU range of 0.5 to 0.95 to capture performance across varying levels of overlap.

### Comparison with state-of-the-art

Table 2 presents the detection results for the UBPD dataset obtained by comparing the proposed TDSS method with several state-of-the-art object-detection frameworks. The comparison included both classic methods such as Mask R-CNN and Cascade R-CNN as well as more recent transformer-based approaches such as DINO. The controlled component comparisons in Tables 3, 4, 5 and 6 use Mask R-CNN with ResNet-50 and FPN as the common baseline, while the state-of-the-art methods in Table 2 follow their respective standard architectures. The proposed TDSS method builds on ResNet-50+FPN and integrates novel SEAE and MSF modules.

Mask R-CNN and Cascade R-CNN follow a traditional multistage detection approach, increasing the complexity and computation time. Dynamic R-CNN adapts to changing distributions during training by adjusting the label assignment and regression loss function, which improves the performance but requires manual adjustments. A sparse R-CNN simplifies object detection by eliminating the need for non-maximum suppression, although it struggles with small targets. ViTDet leverages the transformer architecture, achieving high performance but at the cost of high computational complexity.

DINO uses transformers for end-to-end object detection by applying techniques such as contrastive denoising training and hybrid query selection. Although DINO excels in many general computer vision tasks, its performance on medical images leaves room for improvement. The proposed TDSS method, enhanced using SEAE and MSF, effectively addresses these challenges, achieves the highest AP across all four anatomical categories, and surpasses existing methods for detecting smaller objects, such as nerves.

The state-of-the-art segmentation model MedSAM was also employed to evaluate its performance on the UBPD dataset. However, MedSAM did not achieve satisfactory results, which was attributed to the significant domain gap and lack of pretraining on datasets specifically tailored to brachial plexus ultrasound imagery.

In particular, TDSS achieves higher AP for detecting nerves, a crucial feature in ultrasound brachial plexus imaging, reaching 0.407 compared to 0.392 (DINO), 0.380 (YOLOv9), 0.385 (Grounding DINO), and 0.390 (RTMDet). The higher performance at AP75 (0.593 *vs* 0.583 for DINO, 0.575 for YOLOv9, 0.580 for Grounding DINO, and 0.585 for RTMDet) further demonstrates the ability of the TDSS to accurately locate object boundaries, which is attributed to SEAE and MSF. The superior performance of TDSS highlights the effectiveness of its tailored design for ultrasound imaging, particularly for handling small and complex structures, such as nerves.

Although the overall mAP improvement over strong baselines was moderate, the gain was concentrated on clinically important and localization-sensitive metrics. TDSS improved nerve AP from 0.392 with DINO to 0.407, and AP75 from 0.583 to 0.593. As shown in Table  1, nerve targets are predominantly small or medium-sized and often have weak boundaries in ultrasound images. Therefore, these results indicate improved localization of clinically relevant nerve targets, whereas a higher AP75 reflects better high-IoU boundary localization.

### Comparison on feature extraction network

Table 3 illustrates the comparative effectiveness of the proposed TDSS method against various feature extraction schemes within the classical Mask R-CNN framework. The feature extraction network consistently outputs four types of feature maps (C1, C2, C3, C4), each varying in size, which are then fed into subsequent network structures by employing a multiscale feature fusion network using the feature pyramid network (FPN) method.

The findings indicate that transformer-based methods, such as ViT, Swin transformer, and Biformer, outperform convolution-based networks such as ResNet-50 and VGG-19, particularly on the UBPD dataset. Notably, the Biformer excelled in detecting four types of targets, achieving AP scores of 0.562, 0.549, 0.315, and 0.470, with significant efficacy in detecting larger targets such as muscle tissue. This underscores the superiority of the transformer architecture in modeling global information and its reliance on attention mechanisms for global relationship modeling, albeit at the expense of increased computational and memory requirements.

Considering the specific requirements of medical image detection and practical application scenarios, modest modifications to a convolution-based feature extraction network were implemented. These adjustments yielded detection performances roughly equivalent to those of transformer-based networks, with notable improvements in the detection of the other three target types.

### Comparison on multi-scale feature fusion

Table 4 presents a comparison of the proposed TDSS method with various multi-scale feature fusion strategies within the Mask R-CNN framework. All the feature extraction networks employed ResNet-50. Following the input of feature maps of different scales from the feature extraction network, these maps were utilized for multiple scales.

The FPN primarily addresses multiscale issues in object detection by modifying simple network connections to enhance the small-object detection performance without increasing the computational load of the original model. PANet introduced the concept of bottom-to-top secondary fusion and added a bottom-to-top fusion path to the standard FPN architecture, thus demonstrating the benefits of bidirectional fusion, although its implementation remains relatively simple. The feature pyramid transformer (FPT) utilizes transformer architecture to facilitate information interaction across different layers of the feature pyramid, yielding impressive outcomes. The spatial MSF module extracts enhanced semantic information by performing channel and spatial cross-attention between feature maps from different layers. The experimental results indicated that MSF achieved the highest AP scores of 0.545, 0.523, 0.317, and 0.439 for arteries, veins, nerves, and muscle tissues, respectively. These results substantiate the effectiveness of MSF in integrating multi-scale semantic information and significantly enhancing the multi-scale semantic representation capabilities of the model.

### Evaluation of plug-and-play modules

Table 5 presents the performance improvements achieved by integrating the SEAE, MSF, and SDAD modules into the Mask R-CNN framework. Each module was individually applied to a baseline Mask R-CNN model using ResNet-50 as the feature-extraction backbone and FPN for multiscale feature fusion. To distinguish the independent contribution of each module from their cumulative effect, Table  5 reports plug-and-play experiments in which one proposed module is inserted into the same baseline model. The progressive integration results for TDSS are reported separately in Table  6.

The SEAE module enhances feature extraction by leveraging edge detection algorithms to emphasize the boundaries of anatomical structures. By focusing on both local edge details and the global spatial context, SEAE improves the detection accuracy of small and complex structures such as nerves and vessels. When integrated into the baseline Mask R-CNN framework, SEAE improves the mAP from 0.395 to 0.414, corresponding to an absolute gain of 0.019, i.e., 1.9 percentage points.

The MSF module strengthens multi-scale feature representation by applying channel and spatial cross-attention mechanisms. This allows for a more effective integration of high-level semantic information across scales, addressing the inherent challenges of detecting objects of varying sizes in ultrasound images. When plugged into the baseline model, MSF improves the mAP from 0.395 to 0.416, corresponding to an absolute gain of 0.021, i.e., 2.1 percentage points.

The SDAD module refines the bounding box localization by employing self-distillation strategies and multiple-instance learning. This module is particularly effective in reducing bounding box drift and improving object localization in noisy environments. Integrating SDAD into the baseline Mask R-CNN improves the mAP from 0.395 to 0.402, corresponding to an absolute gain of 0.007, i.e., 0.7 percentage points.

These results collectively substantiate the modularity and effectiveness of SEAE, MSF, and SDAD, showing their ability to independently improve detection performance when seamlessly integrated into existing object-detection frameworks.

### Ablation study

Table 6 presents the ablation experiments that evaluated the impact of the individual components within the TDSS framework. By incrementally integrating these components and examining their effect on AP in the four anatomical structures, the experiments demonstrate how each contributes to the model’s performance.

Together with the plug-and-play evaluation in Table 5, this progressive setting clarifies both the independent utility of each module and their cumulative contribution within TDSS.

The SEAE module enhances the model’s ability to detect targets by accentuating edge features in ultrasound images. It focuses on local edge details and analyzes the imaging differences across biological structures, thereby capturing global channel dependencies. This is particularly beneficial for brachial plexus ultrasound images, where the neural tissue edge features are typically less distinct. By improving edge detection, SEAE helps the model identify the contours of nerve structures more accurately, leading to better detection outcomes.

The spatial MSF module leverages a multi-scale pyramid structure to integrate the feature map information at various resolutions, allowing the model to simultaneously address detailed and global image features. This approach enhances the capacity of the model to understand and represent the interdependencies among features at different scales. Notably, improvements in AP were observed across arteries, veins, nerves, and muscle tissues, with increases of 0.3, 0.6, 0.4, and 0.6 percentage points, respectively. These results demonstrate that MSF effectively captures both global and detailed image characteristics.

The SDAD module addresses the challenge of using inaccurate bounding boxes in noisy ultrasound images. By harnessing self-distillation technology and utilizing stable classification data, SDAD optimizes bounding box positioning based on the classification results used as guidance signals. When integrated with other modules, SDAD improves model performance, achieving AP scores of 0.712, 0.691, 0.407, and 0.531 for arteries, veins, nerves, and muscle tissues, respectively. This indicates an improved ability to locate anatomical structures accurately under conditions of noise interference.

Ablation experiments demonstrate that the SEAE and MSF modules enhance the model’s ability to extract features and understand semantics across varying sizes of anatomical structures in brachial plexus ultrasound images. In addition, the SDAD module reduces noise interference, thereby improving the target positioning capabilities of the model. When combined, these three modules enable a more comprehensive understanding and representation of image information. This integration improves performance on the multi-object detection task for the ultrasonic brachial plexus imaging, enhancing both the accuracy and robustness of the model.

## Conclusions

This study introduced the TDSS model, a novel framework designed to enhance multi-object detection of the brachial plexus in ultrasound images through three novel components: SEAE, MSF, and SDAD. By systematically integrating these modules, the accuracy and robustness of the detection process were improved. Extensive experimental results on the UBPD dataset validate the model’s performance, particularly in overcoming inherent visual degradations, such as speckle noise and low spatial resolution, that commonly affect ultrasound imagery.

In addition to achieving state-of-the-art performance, the three principal modules of the TDSS model contribute valuable methodologies to the broader field of medical image analysis. Specifically, the SEAE module provides a robust mechanism for edge delineation, the MSF module offers a tailored approach for multiscale semantic integration, and the SDAD module introduces an effective strategy for refining localization precision. Their synergistic integration demonstrates that combining low-level structural priors with high-level semantic reasoning significantly improves the effectiveness of transformer-based detection networks.

The practical potential of the TDSS model, as illustrated by the proposed experiments, provides a strong foundation for future research on the clinical integration of ultrasound-guided interventions. Future research will explore the generalizability of the TDSS framework across diverse ultrasound modalities and medical specialties. Furthermore, integrating the model into real-time ultrasound systems could broaden its clinical utility for intraoperative guidance. Considering the high costs and variability associated with medical image annotation, future research will also focus on advancing semi-supervised and unsupervised learning techniques to reduce reliance on extensively annotated datasets.

## Data Availability

The source code for the TDSS framework is publicly available at https://doi.org/10.5281/zenodo.18824199. The datasets analyzed during the current study are available from the corresponding author on reasonable request.

## Abbreviations

PNB: Peripheral nerve block
TDSS: Transformer-based detection with semantic and spatial feature fusion
SEAE: Spatial edge adaptive enhancement
MSF: Multi-scale semantic fusion
SDAD: Spatial distillation-assisted detection
UBPD: Ultrasound Brachial Plexus Dataset
ViT: Vision transformer
DETR: DEtection TRansformer
DINO: DETR with Improved deNoising anchor boxes
FPN: Feature pyramid network
NMS: Non-maximum suppression
IoU: Intersection over Union
MIL: Multiple instance learning
AP: Average precision
mAP: Mean average precision
